# Regulation of Placental Extravillous Trophoblasts by the Maternal Uterine Environment

**DOI:** 10.3389/fimmu.2018.02597

**Published:** 2018-11-13

**Authors:** Jürgen Pollheimer, Sigrid Vondra, Jennet Baltayeva, Alexander Guillermo Beristain, Martin Knöfler

**Affiliations:** ^1^Department of Obstetrics and Gynaecology, Medical University of Vienna, Vienna, Austria; ^2^British Columbia's Children's Hospital Research Institute, Vancouver, BC, Canada; ^3^Department of Obstetrics and Gynecology, University of British Columbia, Vancouver, BC, Canada

**Keywords:** placental development, extravillous trophoblast, decidual immune cells, trophoblast invasion, uterine natural killer cells, decidual macrophages

## Abstract

During placentation invasive extravillous trophoblasts (EVTs) migrate into the maternal uterus and modify its vessels. In particular, remodeling of the spiral arteries by EVTs is critical for adapting blood flow and nutrient transport to the developing fetus. Failures in this process have been noticed in different pregnancy complications such as preeclampsia, intrauterine growth restriction, stillbirth, or recurrent abortion. Upon invasion into the decidua, the endometrium of pregnancy, EVTs encounter different maternal cell types such as decidual macrophages, uterine NK (uNK) cells and stromal cells expressing a plethora of growth factors and cytokines. Here, we will summarize development of the EVT lineage, a process occurring independently of the uterine environment, and formation of its different subtypes. Further, we will discuss interactions of EVTs with arteries, veins and lymphatics and illustrate how the decidua and its different immune cells regulate EVT differentiation, invasion and survival. The present literature suggests that the decidual environment and its soluble factors critically modulate EVT function and reproductive success.

## Introduction

Development of the human placenta, its distinct epithelial trophoblast subtypes and their interplay with maternal cells and growth factors of the pregnant uterus are crucial for a successful pregnancy. After implantation the trophectoderm, the outermost cell layer of the blastocyst, gives rise to mononuclear cytotrophoblasts (CTBs) forming placental villi through branching morphogenesis. During the first weeks of gestation primary villi, consisting of proliferative CTBs, transform into secondary mesenchymal villi and mature tertiary villi, the latter undergoing vasculogenesis and angiogenesis (1–3). At term these tree-like structures of the human placenta display a surface area of ~15 m^2^, completely covered with multinuclear syncytiotrophoblasts (STBs). STBs are generated by cell fusion of villous CTBs (vCTBs) and fulfill a vast range of functions such as production of pregnancy hormones, transport of oxygen and nutrients from the maternal blood stream to the growing fetus and clearance of fetal waste products (4, 5). However, early placental development and fetal growth occurs in the absence of maternal blood and oxygen and are likely supported by growth factors and proteins secreted from endometrial glands (6). As soon as the utero-placental circulation is established between 10th and 12th week of pregnancy placental villi are bathed in maternal blood, and hence are termed floating villi.

**Figure 1:**
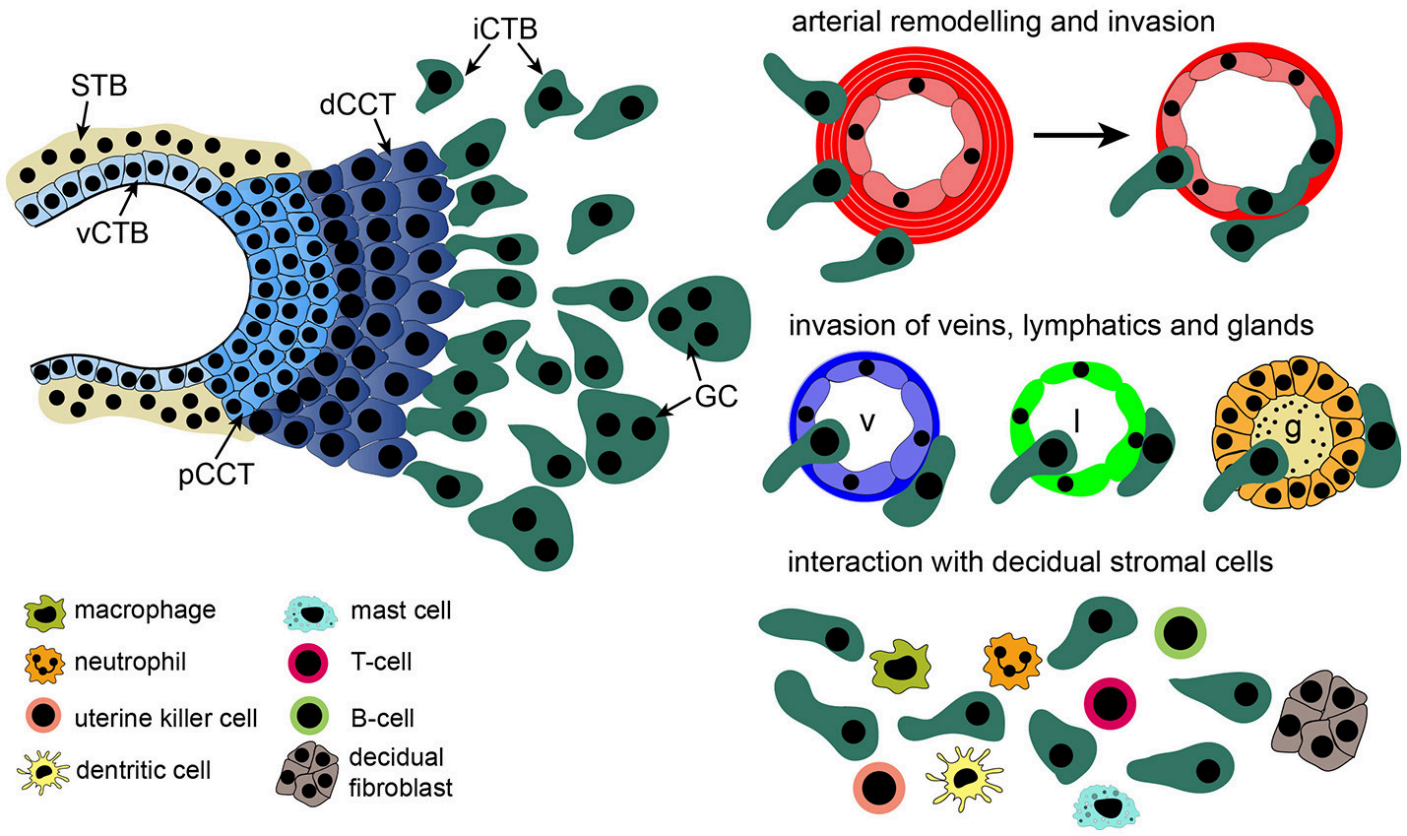
Structure of a placental anchoring villus and its different trophoblast subtypes. Precursors, residing in the villous cytotrophoblast (vCTB) layer either differentiate into multinuclear syncytiotrophoblasts (STBs), surrounded by maternal blood, or give rise to proliferative proximal cell column trophoblasts (pCCTs) upon attachment of villi to the maternal decidua. After differentiation into distal cell column trophoblasts (dCCTs) extravillous trophoblasts (EVTs) develop, breaking through the overlying STB layer. EVTs detach from distal cell columns, migrate into the decidual stroma and the maternal spiral arteries replacing maternal endothelial cells. In the decidual stroma interstitial CTBs (iCTBs) interact with macrophages, B- and T-cells, mast cells, dendritic cells, neutrophils, decidual stromal cells, and uterine natural killer (uNK) cells. Moreover, iCTBs approach the vessel walls of spiral arteries and promote remodeling from outside. Also, veins (v), lymphatics (l) and glands (g) are invaded by these cells forming multinucleated trophoblast giant cells (GC) as an end-stage of differentiation.

Whereas, STBs of floating villi represent the transport units of the human placenta, anchoring villi of the placental basal plate form another differentiated trophoblast type, the so called invasive extravillous trophoblast (EVT). Upon attachment of villi to the maternal decidua, the endometrium of the pregnant uterus, proliferative proximal cell column trophoblasts (pCCTs) develop which further differentiate into distal CCTs (dCCTs) ceasing their mitotic activity (Figure 1). EVTs are formed upon detachment from the distal cell column. These cells deeply migrate into the maternal decidua and the first third of the underlying myometrium (7). Already 2 weeks after fertilization two types of EVTs can be discerned within the maternal uterine compartment, interstitial CTBs (iCTBs), colonizing the decidual stroma, and endovascular CTBs (eCTBs), penetrating the maternal spiral arteries (8). Stepwise modification of these vessels is regarded as a critical step in placentation. In the first weeks of pregnancy, EVTs plug the spiral arteries, likely to prevent precocious onset of blood flow to the developing placenta, hence protecting against early placental damage through oxidative stress and fetal loss (6, 9). However, as the embryo switches from histiotrophic to haemotrophic nutrition after the 10th week gestation, plugs dissolve and the endothelial layer of the spiral arteries is replaced by eCTBs (8, 10). The latter are thought to arise by luminal migration into the myometrial segments of spiral arteries (11). Moreover, iCTBs accumulate in the muscular vessel wall promoting its elastolysis and degradation, where decidual macrophages and uterine natural killer cells (uNKs) also contribute to this process (12). Notably, uNK cells, increasing in numbers during the secretory phase of the menstrual cycle and early pregnancy (13), initiate remodeling by inducing apoptosis of vascular smooth muscle cells, whereas iCTBs are thought to complete this process (14). These modifications transform the spiral arteries into highly dilated vessels ensuring low-pressure blood flow to the placenta and the developing fetus. Both iCTBs and eCTBs upregulate adhesion molecules mimicking an endothelial phenotype which could be instrumental during invasion and for the replacement of maternal endothelial cells (15). Defects in vessel remodeling, in particular in the myometrial part of the spiral arteries, have been reported in various pregnancy complications, such as preeclampsia, fetal growth restriction, preterm labor, abortions, and stillbirth (9, 10, 16–19). Failures in immunological acceptance of the placenta, decidual function and/or abnormal trophoblast invasion and differentiation could be underlying causes (20–23). During the first weeks of gestation EVTs, originating from the trophoblastic shell (24), also migrate into decidual lymphatics and veins, already before arterial remodeling occurs (25–27). Number of EVTs in lymphatic and venous vessels is lower in recurrent abortions suggesting that, along with defects in spiral artery remodeling, failed interactions of EVTs with other types of uterine vessels could contribute to pregnancy complications (26). Similarly, EVTs also invade into the decidual glands which could promote early histiotrophic nutrition (28).

In the decidua basalis, iCTBs communicate with diverse cell types of the fetal-maternal interface, such as decidual stromal cells (DSCs) and different immune cells (Figure 1). Amongst those, uNK cells and macrophages have been delineated as the most abundant cell types (29). The role of uNK cells has been extensively investigated throughout the years. Besides their role in the immunological tolerance of the semi-allogenic fetus, uNK cells are thought to affect decidual angiogenesis and EVT function (22, 30). Expression of human leukocyte antigen C (HLA-C) on EVTs, interacting with killer cell immunoglobulin-like (KIR) receptors on uNK cells, could play a role in pregnancy outcome as certain combinations of fetal HLA-C and maternal KIR alleles might increase the risk of developing preeclampsia and recurrent miscarriage (31, 32). It is anticipated that unfavorable HLA-C/KIR interactions impair trophoblast invasion and as a consequence spiral artery remodeling. The role of KIRs and their effects in allorecognition of EVTs has been subject of numerous reviews (22, 33–36) and will be only briefly discussed herein. Instead, we review how uNK cells influence EVTs in a paracrine manner. Further, we will also focus on the other maternal cell types of the decidua and summarize how they might affect cell column growth of anchoring villi, EVT formation and motility. Factors secreted by EVTs, controlling trophoblast migration and invasion in an autocrine manner, have been extensively discussed elsewhere (37, 38), and will not be presented herein. Likewise, the paracrine effects of EVT-secreted factors on decidual immune cell function will not be a topic of this review.

## Development of the Evt lineage and its different subtypes

EVTs originate from distal cell columns of anchoring villi at distinct contact sites with the maternal decidua. Numbers of the latter are a consequence of the frequency of villous branching (5). Different to growth of vCTBs, that form a double-rowed epithelium after lateral cell division and fuse into STBs, pCCTs break through the overlying STB layer and form multiple layers of proliferative trophoblasts (Figure 1). Similar to early phases of tumor formation, pCCTs detach from the basal membrane and lose their polarity. However, in contrast to cancer cells, growth and invasion of trophoblasts is highly organized and precisely controlled in a spatiotemporal manner. At distal sites of anchoring villi, pCCTs differentiate into non-proliferative dCCTs. Similar to iCTBs which have deeply migrated into the decidua, dCCTs express numerous EVT markers such as HLA-G (39), T-cell factor 4 (TCF-4) (40), integrin α5 (ITGA5) and β1 (41), Notch2 (42), proteoglycan 2 (26). and ErbB2 (43). Hence, formation of dCCTs represents the first step of EVT differentiation. In comparison to dCCTs, iCTBs undergo further differentiation by inducing/upregulating specific proteins, for example ITGA1 (41), matrix metalloproteinase (MMP) 12 (44, 45) or diamine oxidase (DAO) (46). The latter is predominantly expressed in EVTs surrounding decidual vessels and was shown to be decreased in serum samples of early-onset preeclamptic women (46). *In vivo*, DAO is only detected in ~20 and 45% of iCTBs and perivascular CTBs, respectively, providing some evidence for the existence of different iCTB subtypes. Similarly, different EVT populations, identified by single-cell RNA-Seq, have recently been suggested (47). However, it remains largely unknown if variations between iCTBs are specified by the intrinsic genetic program of the placental anchoring villus or determined by the diverse decidual structures. Likewise, the exact route of eCTB migration and the mechanisms specifying these cells have not been unraveled (11). The different phenotypes of EVTs could eventually be influenced by the decidual environment. For example, it was shown that abnormal gene expression of preeclamptic CTBs was reverted back to normal physiological levels when cultured *in vitro* (21). On the other hand, EVT development *per se* occurs independently of the decidual environment and its growth factors. Purified CTBs and villous explant cultures, seeded on extracellular matrix, undergo spontaneous EVT differentiation upregulating dCCT, and iCTB markers in a kinetic manner (48–50). In preeclampsia this endogenous EVT differentiation program could be disturbed (51). Anchoring villi and detaching EVTs of tubal pregnancies show the same pattern in integrin switching as EVTs invading the decidua basalis (52). Similarly, EVTs migrating from implanted villous explants and invading the kidney capsule of SCID mice, were shown to induce HLA-G expression (53).

Although the genome-wide expression profiles of non-migratory CTBs and invasive EVTs have been unraveled (54, 55), mechanisms promoting cell column formation and CTB commitment toward the EVT lineage have been poorly elucidated. Recently, Notch1 has been detected in a subset of proliferative pCCTs, indicating that this particular receptor could mark EVT progenitors (56). Indeed, the active Notch1 intracellular domain promoted pCCT survival and marker expression, but suppressed stemness markers of vCTBs suggesting that Notch1 could convert CTB precursors into EVT progenitors (57). Low oxygen levels, occurring during early phases of placental development (58), were shown to trigger Notch1 expression in primary CTBs (57). Hence, low oxygen could promote expansion of EVT progenitors and promote early stages of EVT differentiation and invasion (59). However, the current literature about the specific role of oxygen in trophoblast biology is controversial, has been extensively discussed (60–63), and will not be subject of the present review. Moreover, changes of the self-renewing conditions of long-term expanding 3-dimensional cytotrophoblast organoid cultures promoted outgrowth of Notch1-positive progenitors and EVT formation (64), further supporting the view that development of different trophoblast subtypes is largely determined by the intrinsic differentiation program of the placenta.

## The impact of the decidua on extravillous trophoblasts: general aspects

In a few species, spontaneous uterine transformation commences during the second half of the menstrual cycle. This process, preceding implantation, is exclusively observed in mammals with menstruation and deep, haemochorial placentation, such as humans and higher primates (65, 66). Shortly after implantation the pregnant uterus undergoes dramatic morphological changes including extracellular matrix remodeling, vascularization, increase in uNK cell numbers and secretory activity of glands as well as transformation of stromal fibroblasts into polygonal decidual cells (67). Decidual glands secrete glycoproteins, such as glycodelin A, carbohydrates and other metabolites nourishing the embryo during the first weeks of pregnancy (68–70). During this phase of histiotrophic nutrition glandular cells also produce various growth factors likely promoting early placental development such as leukemia-inhibitory factors (LIF), epidermal growth factor (EGF), vascular endothelial growth factor (VEGF) and endocrine gland-derived vascular endothelial growth factor (EG-VEGF) (69, 71–74). Indeed, EGF (see below) and EG-VEGF were shown to augment proliferation of vCTBs/CCTs in villous explant cultures (75). Similarly, VEGF was shown to stimulate growth of trophoblast cell lines and primary cultures (76). In contrast, LIF may be mainly involved in the regulation of implantation and trophoblast invasion (77–79).

Differentiation of uterine fibroblasts, commonly referred to as decidualization, initiates during the luteal phase of the menstrual cycle and requires the combined action of cAMP and progesterone on the estrogen-primed endometrium (80). Besides secretion of growth- and- invasion-controlling factors (see below) numerous other functions have been assigned to decidual fibroblasts (DFs). For example, DFs secrete enzymes clearing reactive oxygen species (67, 81) and thereby might protect the decidua and/or EVTs from adverse stress response when local oxygen levels rise between 10th and 12th week of gestation. Trophoblast-derived human chorionic gonadotrophin (hCG) could further increase resistance of DFs against oxidative tissue damage (82). DFs also express various extracellular matrix proteins, such as fibronectin, emilin-1, decorin, fibulins, collagens and laminins (83–86), potentially controlling EVT motility by binding to trophoblast-expressed adhesion molecules and receptors (87).

In women with placenta accreta, EVTs excessively invade the maternal uterus, mostly as a consequence of implantation onto or close to a scar after preceding cesarean section. It is anticipated that the local absence of decidua facilitates trophoblast invasion into the underlying myometrium (88, 89). From this pathology, one might conclude that the decidua restricts migration of trophoblasts thereby controlling depth of invasion in a temporal manner and preventing aberrant, tumor-like expansion of the placenta. Indeed, former concepts suggested that trophoblast-derived MMPs, known to promote invasiveness, are counter-balanced by tissue-inhibitors of metalloproteinases (TIMPs) present in the decidua (90, 91). Similarly, decidual plasminogen activator inhibitors (PAI) 1 and 2 could control timing and depth of trophoblast invasion by inhibiting the plasminogen activator (PA) system expressed by migratory EVTs. However, physiology of trophoblast invasion is more complex since both EVTs and DFs express MMPs, TIMPs, PAs, PAIs as well as urokinase plasminogen activator receptor (uPAR) (90, 92–98). Hence, the decreasing rate of EVT invasion during pregnancy cannot be merely explained by the reciprocal expression of MMPs/uPA and TIMPs/PAI in EVTs and the decidua, respectively. Moreover, potential changes of inhibitor expression throughout gestation or in superficial vs. deeper regions of the decidua have not been measured. Diminished EVT migration at later stages of pregnancy might also be a consequence of the decreasing growth rates of cell columns. As follows, defects in invasion/remodeling, as observed in IUGR, could at least partly be the result of reduced trophoblast growth in this condition (99).

Other data do not support the concept that the decidua restricts trophoblast invasion. In contrast to anchoring villi of normal uterine pregnancies, distal cell columns of ectopic placental villi, isolated from tubal pregnancies, were extended in size. This suggests that the decidua could facilitate EVT detachment from anchoring villi during physiological development of the placental basal plate (52). Indeed, decidualized endometrial stromal cells express a tissue-specific variant of fibronectin favoring trophoblast invasion (100). Whereas, EVTs, generated by first trimester villous explant culture, migrated superficially on dermal fibroblasts, their co-culture with DFs resulted in promotion of interstitial invasion (100). Therefore, the specific features of the decidua may adapt to different stages of pregnancy and precisely control invasion of EVTs by expressing pro- and anti-migratory matrix proteins and factors (37, 38). In return, DFs have a high migratory capacity and could promote implantation by actively moving toward the blastocyst and provoking encapsulation of the conceptus (101). Indeed, EVT supernatants contain chemotactic signals that promote endometrial stromal cell migration (102). Genome-wide expression analyses revealed that trophoblast-conditioned medium of cultivated CTB preparations, containing a mixture of different CTB subtypes, could induce mRNAs encoding chemokines and angiogenic factors in decidualized endometrial fibroblasts (103). However, compared to EVTs, mixed CTB isolates may elicit different responses. In a recent study EVTs increased numbers of resting FoxP3-positive regulatory T cells (Tregs) upon co-cultivation with CD4^+^ T cells, whereas vCTBs were ineffective (104).

## Caveats of *in vitro* studies with extravillous trophoblasts

Decidua-derived growth factors and their role in trophoblast motility have been investigated in numerous publications. However, many of these studies have drawbacks limiting their scientific value. Access to primary trophoblasts of early pregnancy is generally restricted, hence different trophoblast-like cell lines were utilized in invasion and migration assays. Yet, the specific origin of these cell lines is uncertain and their genome-wide gene expression profiles and HLA status differ considerably from purified CTBs (105, 106). Cell lines proliferate in culture whereas EVTs are non-mitotic cells. Hence, discordant results were obtained between cell lines and primary cells, for example in migration assays under hypoxic conditions (107, 108). Similarly, transforming growth factor β (TGFB), expressed by DFs, uNK cells and uterine glands (109, 110), was shown to either promote or inhibit trophoblast proliferation or invasion (111–115). Besides divergence between cell lines and first trimester CTBs, contaminations with highly migratory placental fibroblasts and variations between different cell isolations and primary model systems might account for multiple discrepancies and the high variances observed in trophoblast-related studies. Invasion assays are often performed with pooled fractions of trypsinized primary CTBs representing a mixture of CCTs, EVTs and vCTBs. The latter also invade through 8 μm transwells *in vitro*, whilst undergoing cell fusion *in vivo*. Moreover, nuclear size is a limiting factor in invasion/migration assays (116). Indeed, EVTs become polyploid during differentiation displaying increased nuclear diameter (117, 118). Therefore, EVTs hardly pass membranes with 8 μm pores (50), a fact which has not been considered by the majority of trophoblast invasion/migration studies. Moreover, the high complexity of decidual cell types cannot be mimicked *in vitro*. In addition, local concentrations of soluble factors in the tissue and their variations during pregnancy and between uterine cell types are poorly studied. Therefore, *in vitro* assays are usually performed with saturating levels of recombinant factors. As follows, the prime target cell of many decidual proteins remains uncertain since the respective receptors have been identified on several uterine cell types. As a consequence, opposing roles for particular factors were suggested. For example, IL10 was shown to directly impair CTB invasion, but also to abolish the adverse effects of LPS-treated macrophages on trophoblastic cell migration (119, 120). Additionally, results obtained with first trimester villous explant cultures recapitulating attachment, outgrowth and EVT migration *in vitro* (121) are often interpreted differently by authors. For example, depending on the specific analyses, outgrowth was suggested to be indicative of both increased trophoblast motility and elevated proliferative capacity.

Herein, we focus on the abundant decidual factors, cytokines, and chemokines which have been most convincingly proven to affect EVT formation and function in reliable trophoblast *in vitro* model systems. However, these studies should also be interpreted in the light of the above-mentioned limitations.

## Regulation of extravillous trophoblasts by decidual fibroblasts

During decidualization fibroblasts upregulate key markers of the pregnant endometrium of which prolactin (PRL) and insulin growth factor binding protein-1 (IGFBP-1) are amongst the most abundantly expressed proteins (122–124). Both proteins likely exert pleiotropic effects on different uterine cell types including regulation of decidualization and EVT migration (125–128). Although PRL, also involved in differentiation of the decidual glandular epithelium (129), was shown to promote motility of first trimester CTBs (130, 131), the role of IGFBP-1 is less clear due to the high complexity of the IGF/IGFBP system. Both migration-activating and -inhibiting effects were attributed to IGFBP-1 upon binding to the EVT-expressed fibronectin receptor ITGA5B1 (132–134). However, one of the main functions of IGFBP-1 could be the regulation of IGF bioavailability at the fetal-maternal interface, possibly triggered by EVT-derived IGF-II (135). Upon secretion of IGF-II from these cells decidual IGFBP-1 might get dephosphorylated and further cleaved by EVT-specific MMP-3 and MMP-9 thereby increasing unbound IGFs, the latter stimulating trophoblast migration (126, 136–139). Yet, proteolytic fragments, generated by trophoblast-derived MMPs, may also restrain trophoblast invasion. Endostatin, a cleavage product of decidual collagen XVIII, was shown to impair IGF-II-induced EVT-motility (50, 140).

Besides the prime markers IGFBP-1 and PRL, other classes of soluble DF-secreted factors were suggested to control EVT motility including chemokines, cytokines and ligands of the EGF and Wingless (WNT) signaling pathways (38, 141). Different CXCL and CCL chemokines have been identified in DFs. Their respective receptors are present on uterine leukocytes and EVTs suggesting a role in immune cell trafficking as well as trophoblast migration, respectively (142, 143). For example, CXCL14 was shown to reduce invasiveness (144), whereas CXCL12 promoted CTB migration and suppressed apoptosis of term trophoblasts through its receptor CXCR4 (145–147). CCL2, expressed by DFs, macrophages and EVTs (148, 149), may recruit T helper 17 cells into the decidua, and interleukin (IL) 17 expressed by these cells could promote trophoblast growth and invasion (150). Other interleukins, shown to stimulate CTB invasion, are IL1B (151, 152) and IL8, secreted from uterine NK cells and DFs (153, 154), whereas IL11 had inhibitory effects (155).

While EGF and heparin-binding EGF (HB-EGF), expressed by the decidua, were shown to stimulate trophoblast invasion and outgrowth from villous explants cultures, proliferation of primary EVTs and trophoblastic HTR-8/SVneo cells was unaffected (156–159). Recently, however, we could demonstrate that these factors increased proliferation of vCTBs and CCTs in villous explant cultures of early placentae (160). Moreover, in contrast to vCTBs and CCTs, EVTs largely lack the EGF/HB-EGF-specific receptors EGFR and ErbB4, and induce ErbB2 and ErbB3 during differentiation (43, 161). Heterodimers of ErbB2 and ErbB3 were shown to interact with neuregulin 1, expressed by DFs, protecting EVTs from apoptosis and thereby retaining their differentiation program (43). Therefore, upregulation of EVT invasion/differentiation by EGF/HB-EGF could largely be a consequence of increased CCT proliferation, while direct effects of these factors on EVTs might be negligible.

Like in other developing tissues WNT signaling has been suggested to play a pivotal role in placental morphogenesis and differentiation (141, 162). Activation of the pathway by secreted ligands stabilizes the key mediator of WNT signaling, β-catenin, and promotes its nuclear recruitment (163). In the nucleus β-catenin binds to DNA-binding proteins of the T-cell factor (TCF) family thereby inducing TCF-mediated gene transcription. Invasive trophoblasts, the secretory endometrium and first trimester DFs express a variety of WNT ligands suggesting autocrine as well as paracrine effects of the particular pathway (164, 165). EVT formation and differentiation is strongly associated with activation of canonical Wnt signaling and nuclear expression of β-catenin, TCF-3 and TCF-4 (40, 166). Indeed, migration and differentiation of EVTs requires TCF-4, whereas survival and proliferation of CCTs is induced by WNT5A involving non-canonical mitogen-activated protein kinase (MAPK) activity (166, 167). Moreover, canonical Wnt signaling might play a dual role in early placental development controlling both long-term expansion of vCTB progenitors and EVT differentiation (64).

## Immune cell distribution in the decidua

The pregnant uterus is mainly colonized by cells of the innate immune system, of which the most abundant and by far best characterized cell types are macrophages and uNK cells. Most available literature refers to numbers ranging from 50 to 70% uNK cells, 20–30 % macrophages and 10–15 % T cells of the total CD45^+^ immune cells in the decidua; only 2 % account toward the less abundant leukocyte populations including dendritic cells or Tregs (30, 168–170). However, the vast majorities of comparative analyses do not consider regional differences (parietalis vs. basalis) in decidual immune distribution and may also miss certain cell populations due to pre-selective isolation methods or due to the lack of appropriate markers to distinguish certain immune cell populations from each other. Hence, before describing uNK cell and macrophage function in detail, we will shortly discuss decidual immune cell populations which in our opinion have widely been ignored in the context of reproductive biology.

Mast cells have been previously described to mainly colonize the uterine myometrium and were shown to localize around decidual vessels. Interestingly, mast cell depletion in a mouse model results in diminished spiral artery remodeling and as a consequence leads to IUGR. The same study demonstrated a close spatial distribution of mast cells and EVTs in the human decidua basalis and reported a mast cell-dependent positive effect on EVT migration (171). Another study showed a role for neutrophils in placentation and trophoblastic giant cell invasion in mice (172). Interestingly, neutrophils seem to accumulate around spiral arteries and develop a pro-angiogenic phenotype toward the second trimester of pregnancy (173). The reason for the oversight of neutrophils might be explained by methodological issues as most protocols to obtain tissue leukocytes involve density gradient centrifugation eliminating all non-mononucleated immune cells, including neutrophils. Nevertheless, it should be taken into consideration that neutrophil accumulation in the decidua may at least be partly a response to blood coagulation and tissue damage occurring during tissue collection. Although mostly ignored, some scientific papers describe the presence of B cells in human term decidua (174). Moreover, a recent study shows that term decidua basalis contains more B cells when compared to decidua parietalis tissues (175). The authors of this study further found that decidua parietalis contains a higher proportion of mature/naive B cells whereas transitional B cells were enriched in decidua basalis. Since altered B cell distributions have recently been associated with preterm labor (174), more studies are needed to determine the role of B cells during pregnancy. In addition, distribution and characterization of B cells in first trimester decidua tissues has not been studied so far. While macrophages are considered to be the main phagocytic and antigen-presenting cell type in the human decidua little is known about dendritic cell distribution and function during pregnancy. One reason for the scarce information concerning decidual dendritic cells is the lack of marker combinations, which could reliably segregate macrophages from dendritic cells, since they develop from a common myeloid progenitor and therefore express common cell surface markers (176, 177). A good example for the problem to distinguish between macrophages and dendritic cells are Langerhans cells. These cells have long been referred to as long-lived dermal dendritic cells and are now considered tissue-resident macrophages with features of dendritic cells such as T cell-stimulation in lymph nodes (178). Despite overlapping cell marker expression and functional similarities in the skin, dendritic cells have unique properties. For instance, DCs homeostatically migrate to draining lymph nodes and are much more potent in antigen cross-presentation to CD8^+^ T cells (179). Consequently, DCs are likely involved in shaping host immune responses toward the invading EVTs.

## Proposed functions of uterine natural killer cells

Unlike conventional peripheral blood (pb) NK that are efficient killers, uNK cells in rodents and humans do not normally mount cytotoxic responses against fetal or placental tissues. Instead, growing evidence highlights the importance of uNK in controlling uterine neo-angiogenesis, spiral artery remodeling, the immune response against fetal antigen, and trophoblast function (30, 180–182). However, recent work shows that aberrant inflammation in pregnancy resulting from infection or fetal-driven alloimmunity programs uNK cells to acquire cytotoxic properties that promote fetal death and/or placental dysfunction (183, 184). Therefore, a contemporary view suggests that appropriate uNK activation is important for promoting healthy placentation, where *inadequate* (not enough) or *inappropriate* (too much) uNK activity contributes to defective placentation and related disorders of pregnancy that may include recurrent miscarriage, preterm birth, and preeclampsia (185, 186). In women, uNK cell numbers rapidly expand during the progesterone-dominant luteal phase of the menstrual cycle (22). Evidence suggests that the decidual environment, enriched with factors like progesterone and transforming growth factor TGFB1, promote the differentiation of NK cell progenitors into mature uNK cells that are defined phenotypically as CD56^superbright^/CD16^−^ cells (187, 188). By contrast, the phenotype of conventional pbNK cells is predominantly CD56^dim^/CD16^+^. Other distinctive features of uNK include the expression of tissue-residency markers (i.e., CD9, CD69, CD49a) (189, 190) and cytolytic proteins (i.e., perforin, granzyme, and granulysin) (191), and the expression of a distinctive natural killer receptor repertoire (192). In particular, killer immunoglobulin-like receptor (KIR) and natural killer group 2 (NKG2) receptors are robustly expressed by uNK cells (189, 192). These and other natural killer cell receptors, are thought to modulate maternal-fetal recognition, but may also play important roles in controlling aspects of trophoblast biology.

## Communication between invasive trophoblasts and uterine natural killer cells

*In vivo* evidence shows that EVTs directly interact with uNK cells (193), indicating that by EVT- uNK cell interactions these potentially modulate each other's functions (194–196). EVTs, unlike vCTBs that do not express major histocompatibility complex (MHC) type-I molecules, express a unique combination of classical HLA-C and non-classical HLA-E, HLA-F, and HLA-G class-I ligands playing a role in immunological acceptance of the placenta/fetus (30, 197, 198). This unique MHC composition enables EVT to directly interact with and modulate uNK cell processes through specific combinations of natural killer receptors. However, hard evidence for *in vivo* or *ex vivo* natural killer cell receptor-EVT interactions has been challenging to generate, due in large part to the ethical boundaries of working with human samples of pregnancy and to the logistical hurdles of working with primary CTB cultures and uNK cells isolated from tissues of the same pregnancy. As a surrogate for primary EVTs, co-cultivation of trophoblastic HTR-8/SVneo cells with uNK cells were performed demonstrating elevated uNK cell survival and downregulation of the activating NKG2D receptor (199). However, these data have to be interpreted with caution since HTR-8/SVneo cells express a different repertoire of HLA proteins, including HLA-A and HLA-B, which are absent from EVTs (105).

MHC class-I molecules expressed on EVTs can interact with multiple natural killer receptors that transmit inhibitory or activating signals to dampen or promote uNK cytotoxicity and production of cytokines, respectively (30). Perhaps the best-studied uNK cell receptors are the family of polymorphic KIRs that are defined by the presence of either 2 (2D) or 3 (3D) immunoglobulin-like domains and long (L) or short (S) cytoplasmic tails that help initiate inhibitory (L) or activating (S) signals. Inhibitory KIRs expressed on uNK cells include KIR2DL1, KIR2DL2, and KIR2DL3, and these receptors transmit strong inhibitory signals through their immunoreceptor Tyr-based inhibitory motif (ITIM) (35). Activating uNK KIRs include KIR2DS1 and KIR2DS4 (186, 200), however the receptor KIR2DL4, an unconventional KIR that predominantly localizes to endosomes and not the cell membrane, is also capable of transmitting activating signals (104, 201, 202). KIRs bind mainly to HLA-C, expressed by multiple cell types within decidual tissue, including EVTs (203). The number of *KIR* genes in the genome of any given individual varies within the population, as does the expression of haplotypic specific HLA-C, making the immunogenic complexities of uNK cell-EVT responses unique for any given pregnancy (204).

To date, most research has examined the importance of HLA-C in controlling uNK cell-related processes through either uNK-target cell or antibody cross-linking experiments. It is important to note that, the directionality of uNK cell response to HLA-C depends largely on the epitope type, designated broadly as C1 or C2. This designation is based on a dimorphism at position 80 of the α1 domain of HLA-C (205). Overall, in mixed uNK cell populations that express high levels of inhibitory KIR, HLA-C challenge promotes an inhibitory signal regardless of the co-presence of activating KIRs (186). KIR-directed inhibitory signals associate with impaired or blunted degranulation (206) and reduced secretion of EVT-regulatory factors (i.e. IL8, VEGF, placental growth factor (PGF) and CXCL10, also known as IP-10) (153). In uNK cells, expressing activating KIR2DS1 or KIR2DS4, HLA-C promotes uNK cell degranulation and secretion of granulocyte-macrophage colony-stimulating factor (GM-CSF) and tumor necrosis factor (TNF) (186, 200, 207). Production of these factors potentially impacts EVT biology (discussed below). However, the direct effect of endogenous HLA-C expressed by EVTs on uNK cell processes has yet to be determined. Nonetheless, *ex vivo* EVTs physically interact with HLA-C-specific KIR2DL1 and KIR2DS1 (204), indicating that biological functions for these KIR-EVT interaction likely do exist.

HLA-E protein is present in EVTs at the 5th week of gestation but absent from these cells after the 7th week, suggesting a predominant role in implantation and/or early trophoblast development (198). Inhibitory signals elicited by HLA-E are mediated through the dimeric CD94/NKG2A receptor (208). Previous work shows that CD94/NKG2A elicits strong suppressive signals that generally override most activating inputs (209). However, whether EVT-derived HLA-E's sole purpose is to restrain uNK cell cytotoxicity in pregnancy is currently not well understood. For example, CD94 function-blocking experiments do not potentiate trophoblast killing (208), suggesting that CD94/NKG2A may serve other roles within the maternal-fetal interface that have yet to be elucidated.

The role of HLA-G in controlling uNK cells has been studied more so than other MHC class-I ligands, due in part to the relevance of its unique and restricted expression within EVT (197). Previous studies have identified two receptors expressed on uNK cells that interact with HLA-G: leukocyte immunoglobulin-like subfamily B member 1 (LILRB1) (210) and KIR2DL4 (211, 212). Interpretation of previous HLA-G-related findings necessitates caution due to the nature and design of the experimental systems used. For example, most approaches implement forced ectopic expression of HLA-G in HLA-null target cell lines, designed to synthesize either of two major HLA-G isoforms expressed by EVTs, namely membrane-bound HLA-G1 (213) or the truncated soluble HLA-G5 (211, 214). Further, many studies have interchangeably used peripheral blood NK cells or NK-like cell lines as surrogate readouts of HLA-G-uNK cell interaction within the maternal-fetal interface. These assumptions have perhaps contributed to over-interpretation of the role of HLA-G in controlling uNK cell-related processes in pregnancy. Conflicting findings have arisen from these studies, indicating that both membrane-bound and soluble HLA-G enhance the production of pro-inflammatory (IFN-γ, TNF, IL1B, IL6) and angiogenic (IL8) factors in endometrial (i.e., not decidual) (213, 215) or pbNK cells (202, 216). In support of the above findings using pbNK cells, Li et al. demonstrated that HLA-G treatment of *ex vivo-*derived uNK cells induces IL6, IL8, and TNF through processes dependent upon KIR2DL4 (212). In contrast to this, *ex vivo* uNK cells, exposed to membrane-bound HLA-G, resulted in inhibition or had marginal impact on uNK degranulation and cytokine production (214). Two recent studies, directly examining the function of HLA-G in primary EVTs and uNK cells, indicated that HLA-G does not induce cytokine secretion from uNK cells but instead dampens uNK cell activity (104, 193), a finding that is consistent with the original paradigm that HLA-G likely promotes immune cell tolerance within the fetal-maternal interface.

## Regulation of extravillous trophoblasts by uterine natural killer cells

Uterine NK are robust producers of cytokines and growth factors, and due to their close proximity to EVTs within the maternal-fetal interface, these cells likely play roles in regulating the diverse functions of trophoblasts. In support of this, uNK cell-generated conditioned media modifies specific biological processes of primary trophoblast, including the promotion of cell invasion (153, 154, 217). Notably, the pro-invasive effect of uNK cell-derived conditioned media is solely attributed to soluble factors produced by uNK cells harvested from later gestational age time-points within the first trimester of pregnancy (i.e., 10–13 weeks' gestation). By contrast, soluble factors produced by uNK cells from the earlier first trimester time-points do not elicit pro-invasive characteristics on EVTs, indicating that the composition of uNK cell factors is influenced by processes related to gestational age, and that the impact of uNK cells on EVT biology is relative to stage of development.

Multiple factors produced by uNK cells have been identified, and importantly, receptors for many of these substances are expressed on primary EVTs. For example, uNK cells produce high levels of IL8, TNF, interferon (INF) γ, TGFB1, CXCL10, as well as the angiogenic factors such as vascular endothelial growth factor A (VEGF-A), VEGF-C and PGF (153). Reciprocally, immunolocalization studies on implantation sites and *ex vivo* studies using primary EVTs provide evidence that invasive trophoblasts populating the maternal-fetal interface produce receptors for these ligands. For example, EVTs express CXCR1 (an IL8 receptor), CXCR3 (an CXCL10 receptor), TNFR1, as well as VEGFR-1 and VEGFR-3, the latter binding VEGF-A and VEGF-C, respectively (110, 153, 218, 219). Notably, supplementation of IL8 and CXCL10 promoted migration of primary CTBs (153). Likewise, inhibition of ligand binding to VEGFR-1 and VEGFR-3 diminished trophoblast invasion (220). Moreover, downregulation of VEGF in uNK cell-conditioned media impaired EVT outgrowth compared to controls (221). In contrast, TNF and IFN-γ inhibited trophoblast migration and invasion by increasing PAI expression and impacting MMP-directed proteolysis, respectively (222, 223). Taken together, factors produced by uNK cells do have the ability to control EVT-related processes *in vivo*. Given the interplay between promoting and restraining invasive characteristics in EVTs, uNK cells could be an important cellular component of the decidua that controls depth of EVT invasion as well as extent of trophoblast-mediated spiral artery remodeling.

## Can the breakdown of maternal-fetal tolerance be related to uterine natural killer cells dysfunction?

Much research related to uNK-trophoblast interactions has centered on the possibility that uNK cells, activated by infection or inflammation, may mount cytotoxic responses toward the semi-allogeneic fetus and trophoblast, thus contributing to infection-related miscarriage and other pregnancy disorders with aberrant inflammation. Surprisingly, most well designed studies, utilizing primary syngeneic uNK cell and trophoblast co-cultures, provided convincing evidence that trophoblasts (both HLA-G- and non-HLA-G-expressing trophoblasts) are highly resistant to uNK-directed killing (193, 224). Although uNK cells do not target trophoblasts for killing, even when artificially activated, uNK still retain pro-cytotoxic features (i.e. granzyme, perforin) that enable efficient cellular immune responses against virally-infected maternal uterine stromal cells, highlighting that trophoblasts are immuno-privileged (193, 225). Nonetheless, research in mice suggests that uNK cells can adopt anti-trophoblast characteristics in the right context. For example, aberrantly activated uNK cells in response to inflammation induced by bacterial endotoxin (183) or alloimmunogenic responses (184) target fetal tissues, including the placenta, and induce fetal resorption. These uNK-driven processes lead to impairments in uterine artery remodeling and placental sufficiency, and can be reversed through genetic ablation strategies (i.e., IL15^−/−^) (183) or antibody-directed inhibition of uNK cells (184). It has also been suggested that uNK cell numbers are altered in pregnancy complications such as PE and IUGR, although contradictory data have been published. Reduced numbers of uNK cells in pregnancies with IUGR have been consistently demonstrated using different methods (226–229). Some studies also suggest a decrease of uNK cells (229) or of the CD56^+^/CD16^+^ uNK subset (228) in PE compared to healthy pregnancies. In contrast, others describe an elevated number of total uNK cells or of the CD56^+^/CD16^+^ subset in PE (230, 231). More recently, research has identified pre-existing health conditions of the mother that associate with low-grade inflammation, like obesity, that potentiate uNK cell activity and modify how uNK cells interact with fetal MHC ligand (207). However, although the *in utero* environment likely shapes uNK cell processes, to date, hard evidence showing that aberrantly activated uNK cells in humans directly target trophoblasts for killing has yet to be clearly demonstrated.

## Regulation of extravillous trophoblasts by decidual macrophages

Next to uterine natural killer cells, macrophages are thought to comprise the second largest leukocyte population within the decidua (168, 232). Besides suggested contributions to spiral artery remodeling and immune modulation (12, 233), the function of decidual macrophages, in particular their effect on EVT activity, remains largely unknown. Although historically described for their function in immune defense, inflammation, and clearance of apoptotic cells, macrophages have also been recognized to play important roles in the development, homeostasis, and repair of various tissues (234).

Macrophages are usually classified into M1, representing a classical pro-inflammatory, anti-microbial activation or into M2, referring to an anti-inflammatory phenotype promoting wound healing. A body of growing evidence however suggests that M1 and M2 rather represent two extreme poles of a broad spectrum of macrophage polarization (235). The non-exclusivity of M1 and M2 macrophage phenotypes *in vivo* has likely several reasons. Firstly, it has been shown that macrophage activation statuses are reversible when specific stimuli change. Secondly, macrophages are often exposed to opposing activating signals *in vivo* (236). For instance, the co-existence of pro-inflammatory M1 and anti-inflammatory M2 profiles has been demonstrated in various mouse models and during tumor progression (237, 238). In light of these data it is not surprising that decidual macrophages also show a unique activation status with a dominating but not exclusive M2 phenotype. While decidual macrophages express typical M2 markers such as CD209 and CD206 and secrete the anti-inflammatory cytokines IL10 and TGFB, they were also shown to secrete pro-inflammatory cytokines including IL6 and TNF and the neutrophil chemoattractant CXCL8 (IL8) (239, 240). Nevertheless, the anti-inflammatory M2-related cytokines IL10 and M-CSF were shown to be important for inducing a decidual macrophage phenotype in peripheral blood monocytes (240). In addition, decidual macrophages were able to suppress T cell activity and induce Tregs *in vitro* (194, 241), which further strengthens the notion of an M2-dominated function. Two independent studies suggested the presence of two distinct decidual macrophage subpopulations, characterized by the absence or presence of the cell surface markers CD11c and ICAM3 (239, 240). Expression of these markers may at least partly relate to immature macrophages or blood contamination, as high levels of CD11c and ICAM3 are also found in blood monocytes. More recently, a study described three decidual macrophage subtypes, based on the expression or absence of CCR2 and CD11c. Using RNA sequencing and functional assays, the authors identified distinct functional states, including differences in phagocytosis, anti-oxidative, and anti-inflammatory activities, and proximity to EVTs (242). Whether these differences indeed account for unique subpopulations or reflect cellular plasticity and thus relate to phenotypical alterations awaits further clarification.

Due to their high abundance in the decidua, it is conceivable that macrophages markedly influence the local paracrine environment and thus EVT function. As follows, it is interesting to note that decidual macrophages are more abundant at the site of implantation and accumulate at the invasive front of EVTs (243, 244). There is growing evidence for a macrophage-guided growth-promoting function in various epithelia (245–247). Interestingly, both placental and decidual macrophages could promote proximal cell column proliferation by secreting the M2-associated (248, 249) factors IL33 and Wnt5a (167, 250). These data suggest that paracrine activity of decidual macrophages could be important for the initial steps in EVT formation. Furthermore, decidual macrophages have been shown to secrete a range of factors known to alter EVT motility (239), albeit with conflicting evidence as to whether they promote or restrict EVT invasion. For instance, while IL8 (153) was described as pro-invasive factor in the context of trophoblast migration, TNF (222) and IL10 (119) have been shown to inhibit EVT motility. The net effect of these factors on EVTs may be pro-invasive, anti-invasive, or neither. In addition, it is unclear under which circumstances decidual macrophages produce these opposing cytokines in terms of macrophage polarization and EVT response. Phenotypic macrophage polarization is controlled by a complex array of soluble factors provided by the local microenvironment and even dictated by extracellular matrix-dependent cell morphology (251). Moreover, ligand distribution within tissues is limited by a wide variety of factors, including limited diffusion capacities, endocytosis, and interaction with extracellular matrix proteins. It is therefore also important to consider the spatial relationship between macrophages and EVTs.

Unfortunately, there is limited information on the difference in macrophage distribution between decidua basalis and parietalis, and especially on whether these different tissue compartments harbor specific macrophage phenotypes. Immunohistochemical studies provided evidence for an enrichment of macrophages in the decidua basalis (243, 244), which has recently been confirmed via flow cytometric analysis (175), suggesting that macrophages preferentially accumulate in the vicinity of EVTs. In a similar context, it has been shown that binding between HLA-G homodimers and macrophage-associated leukocyte immunoglobulin-like receptor B1 (LILRB1) upregulates secretion of IL6, IL8, and TNF (212). Although it is not clear whether these effects relate to membrane-bound or soluble HLA-G, the presence of EVTs likely influences the macrophage phenotype and thus could substantially influence the paracrine activity of decidual macrophages.

## Are decidual macrophages altered in complicated pregnancies?

Complicated pregnancies with IUGR or early-onset PE have repeatedly been associated with compromised EVT function. Several studies have tried to decipher whether aberrations in the decidual macrophage population could mediate these EVT defects, albeit with conflicting results. Some studies suggest an increase in macrophage numbers in preeclampsia compared to healthy pregnancies (252, 253). Further, an inverse relationship between macrophage infiltration and invasion of trophoblasts into arteries, with a shift toward macrophage infiltration in preeclamptic pregnancies, was shown (252). On the contrary, other studies point toward a decrease in the number of macrophages in IUGR and PE, compared to healthy pregnancies (229, 254). Still some additional studies found no significant differences in macrophage distribution and activation patterns between preeclamptic women and preterm labor controls (255).

In addition, it is unclear whether aberrant macrophage polarization could be associated with EVT defects in the development of placental pathologies. In detail, some evidence exists that macrophages could display a more M1-like polarization in cases of PE, resulting in an exacerbated production of pro-inflammatory cytokines adversely affecting EVT function (256, 257). For instance, TNF has been shown to inhibit trophoblast motility in villous explant cultures (222). A study utilizing a rat model, demonstrating that systematic LPS injection results in IUGR and PE-like symptoms, also reported increased levels of TNF and exacerbated numbers of uteroplacental macrophages (258). Unfortunately, the authors did not further elucidate whether LPS-induced systemic inflammation also changes the phenotype of decidual macrophages. Nevertheless, whether a shift toward a pro-inflammatory M1-like macrophage phenotype indeed adversely affects EVT function has not been proven. As mentioned above, M2-related anti-inflammatory cytokines such as TGFB or IL10 were also reported to exert adverse effects on EVTs by restricting their migratory potential. Conversely, macrophages isolated from miscarriages showed reduced expression of IL6 and IL8, the latter with pro-invasive potential toward EVTs (153). In summary, studies investigating the functional interplay between primary macrophage and EVT cultures are scarce. Thus, the particular role of macrophages in the context of EVT function remains unclear both in healthy and complicated pregnancies. Unfortunately, there is also limited availability of suitable human *in vitro* systems to study this interaction. Immortalized monocytic cell lines, such as THP-1 cells, do not represent a useful model system for decidual macrophages due to their massive genomic rearrangements and their phenotypic and functional differences (259). It is still unclear whether the decidua-specific macrophage phenotype can be sustained in isolated primary cells or mimicked by controlled differentiation of peripheral blood monocytes *in vitro*. Moreover, more information is needed on whether macrophages change their polarization and secretory profile depending on their location within the human decidua. Finally, the long lasting paradigm of macrophage differentiation from recruited monocytes has been challenged by numerous studies demonstrating that macrophages are also maintained throughout adult life by a tissue-resident, proliferative population originating from embryonic or yolk sac-derived precursors (260, 261). In mice, tissue-resident macrophage populations, such as liver Kupffer cells (262), epidermal Langerhans cells (263), microglia (264), and pleural macrophages (265), were shown to be able to proliferate and renew independently from the bone marrow. Although very few data have been generated so far to confirm the existence of tissue-resident macrophages in humans, it is interesting to note that decidual, tissue-resident CD34^+^ stromal cells were described to differentiate into functional CD56^+^ uNK cells (266). Moreover, a recent study shows that continuous pregnancies induce a pregnancy-promoting memory uNK subset which differentiates from progenitors residing in the post-gravid endometrium (267). Additional studies, confirming the existence of tissue-resident NK cells and T cells in other tissues, strengthen the idea of an organ-specific immunity that is maintained independently of the bone marrow and secondary lymph nodes (268, 269). Intriguingly, several reports describe the proliferative signature of decidual macrophages (270, 271), supporting the idea that tissue-resident macrophages could be maintained in the uterus by local proliferation. On the other hand, both EVTs and decidual macrophages produce monocyte-recruiting chemokines, such as CCL2 (148, 149) or CCL4 (148, 239) suggesting a contribution of monocytes to the pool of decidual macrophages in early pregnancy. In light of these data it is still not clear whether the endometrium is continuously populated by bone marrow-derived monocytes or whether local progenitors mainly repopulate within the tissue.

## Summary

**Figure 2:**
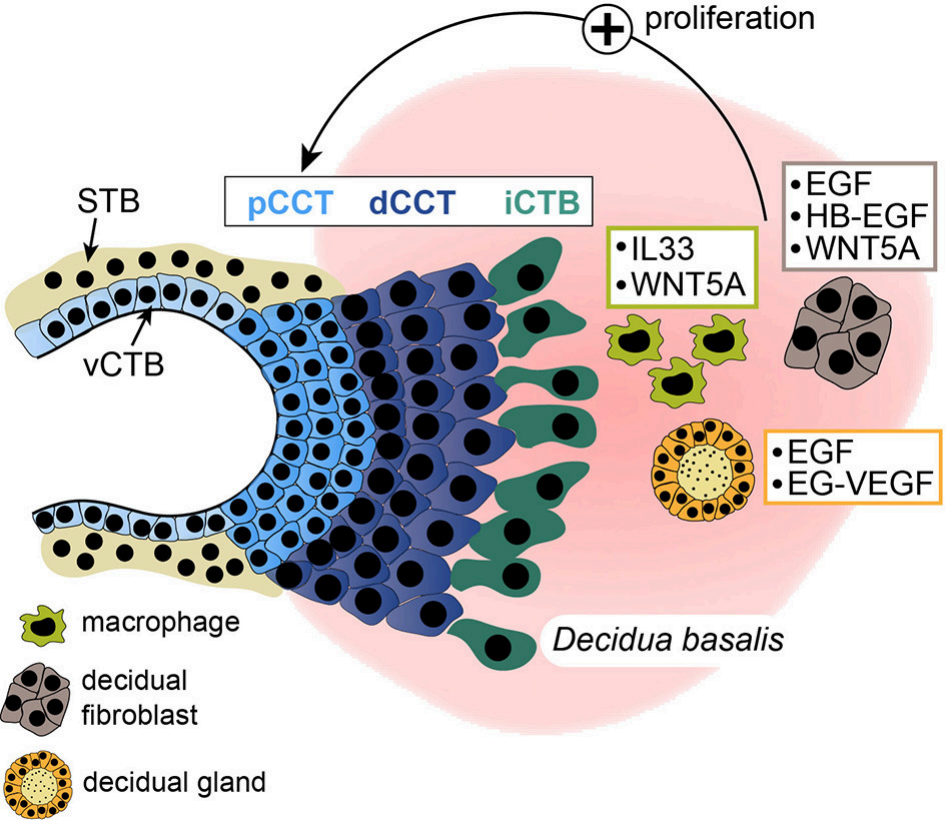
Schematical depiction of soluble factors secreted from decidual macrophages, stromal cells, or glands. Mediators, stimulating proliferation of proximal cell column trophoblasts (pCCTs) in villous explant cultures are illustrated. dCCT, distal cell column trophoblast; STB, syncytiotrophoblast; vCTB, villous cytotrophoblast; iCTB, interstitial cytotrophoblast.

**Figure 3:**
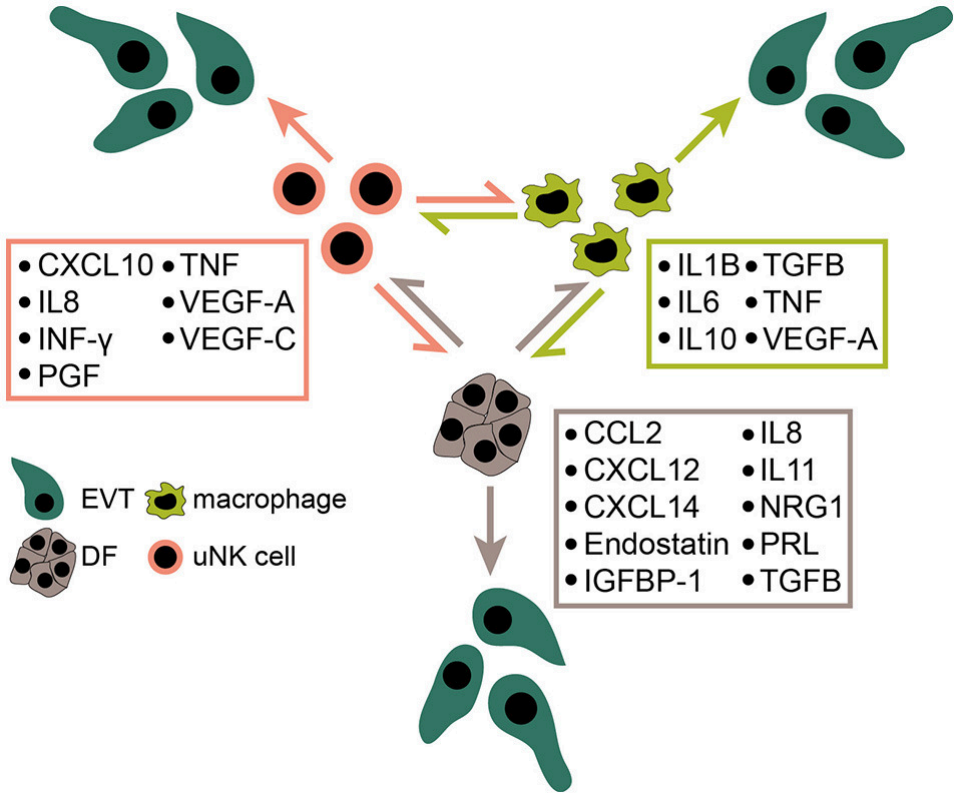
Expression of decidual chemokines, cytokines and other soluble mediators affecting trophoblast migration and/or invasion. Some of the depicted factors likely play numerous roles at the fetal-maternal interface including activation and maturation of immune cells, as well as decidual angiogenesis and spiral artery remodeling. EVT, extravillous trophoblast; uNK cell, uterine natural killer cell; DF, decidual fibroblast.

The influence of soluble decidual factors on trophoblast proliferation, migration and invasion has been intensively investigated using different trophoblast cell models including cell lines and primary CTBs containing a mixture of different trophoblast subtypes. However, studies about the effects of secreted proteins on cell column proliferation of the anchoring villus are scarce. Moreover, isolated primary CCTs rapidly cease proliferation in culture and undergo differentiation (57), impairing their usability for proliferation assays. Hence, only few factors were convincingly shown to promote CCT expansion using first trimester villous explant cultures (Figure 2). Recently however, self-renewing trophoblast stem cells and organoids have been developed (64, 272). These culture systems should allow more reliable investigations on the role of decidual growth factors in trophoblast progenitor growth, EVT formation and differentiation. Furthermore, DFs, macrophages and uNK cells express a plethora of cytokines, chemokines and soluble factors, some of which are detectable in more than one cell type (Figure 3). Although the majority of these proteins likely control trophoblast invasion and/or migration the prime decidual target cell of an individual factor remains elusive. Besides their presumptive role in trophoblast motility, chemokines and cytokines could regulate immune cell recruitment and mutual activation of macrophages, uNK cells and DFs as well as other less abundant immune cells of the fetal-maternal interface.

## Author contributions

The manuscript was written by JP, AGB, and MK and edited by SV and JB. JP provided the graphical illustrations.

### Conflict of interest statement

The authors declare that the research was conducted in the absence of any commercial or financial relationships that could be construed as a potential conflict of interest.
